# Protein Binding of Lapatinib and Its N- and O-Dealkylated Metabolites Interrogated by Fluorescence, Ultrafast Spectroscopy and Molecular Dynamics Simulations

**DOI:** 10.3389/fphar.2020.576495

**Published:** 2020-10-30

**Authors:** Inmaculada Andreu, Emilio Lence, Concepción González-Bello, Cristobalina Mayorga, M. Consuelo Cuquerella, Ignacio Vayá, Miguel A. Miranda

**Affiliations:** ^1^ Departamento de Química/Instituto de Tecnología Química UPV-CSIC, Universitat Politècnica de València, València, Spain; ^2^ Unidad Mixta de Investigación UPV-Instituto de Investigación Sanitaria (IIS) La Fe, Hospital Universitari i Politècnic La Fe, Valencia, Spain; ^3^ Centro Singular de Investigación en Química Biolóxica e Materiais Moleculares (CiQUS), Departamento de Química Orgánica, Universidade de Santiago de Compostela, Santiago de Compostela, Spain; ^4^ Allergy Clinical Unit, Hospital Regional Universitario de Málaga and Allergy Research Group, Instituto de Investigación Biomédica de Málaga-IBIMA, Málaga, Spain

**Keywords:** femtosecond transient absorption, fluorescence, hypersensitivity reactions, lapatinib, metabolites, molecular dynamics simulations, protein binding

## Abstract

Lapatinib (LAP) is an anticancer drug generally used to treat breast and lung cancer. It exhibits hypersensitivity reactions in addition to dermatological adverse effects and photosensitivity. Moreover, LAP binds to serum proteins and is readily biotransformed in humans, giving rise to several metabolites, such as N- and O-dealkylated products (N-LAP and O-LAP, respectively). In this context, the aim of the present work is to obtain key information on drug@protein complexation, the first step involved in a number of hypersensitivity reactions, by a combination of fluorescence, femtosecond transient absorption spectroscopy and molecular dynamics (MD) simulations. Following this approach, the behavior of LAP and its metabolites has been investigated in the presence of serum proteins, such as albumins and α_1_-acid glycoproteins (SAs and AGs, respectively) from human and bovine origin. Fluorescence results pointed to a higher affinity of LAP and its metabolites to human proteins; the highest one was found for LAP@HSA. This is associated to the coplanar orientation adopted by the furan and quinazoline rings of LAP, which favors emission from long-lived (up to the ns time-scale) locally-excited (LE) states, disfavoring population of intramolecular charge transfer (ICT) states. Moreover, the highly constrained environment provided by subdomain IB of HSA resulted in a frozen conformation of the ligand, contributing to fluorescence enhancement. Computational studies were clearly in line with the experimental observations, providing valuable insight into the nature of the binding sites and the conformational arrangement of the ligands inside the protein cavities. Besides, a good correlation was found between the calculated binding energies for each ligand@protein complex and the relative affinities observed in competition experiments.

## Introduction

The human epidermal growth factor receptor (HER) family is composed of four different members that have been thoroughly investigated due to their important role in cancer progression. HER receptors are transmembrane proteins that control a variety of cell functions such as cell differentiation, proliferation, apoptosis, migration and angiogenesis (Nicholson et al., 2001; Hynes and Lane, 2005; Thomas and Weihua, 2019). However, pathological alterations including overexpression or mutations in the tyrosine-kinase site to HER-1 and/or HER-2 are directly associated with the development of different types of human cancer (Hynes and Lane, 2005; González and Lage, 2007; Ross et al., 2016; Sigismund et al., 2018; Thomas and Weihua, 2019).

**FIGURE 1:**
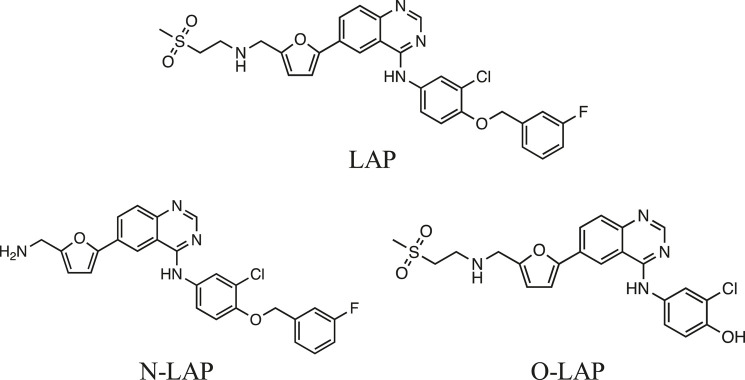
Chemical structures of lapatinib (LAP) and its main metabolites N-LAP and O-LAP.

Lapatinib (LAP) is an orally administered drug that strongly inhibits HER-1 and HER-2. It is generally used to treat breast cancer, but due to its dual HER targeting it is expected to exhibit higher activity than monotargeted tyrosine-kinase inhibitors; in addition, its relatively small size allows LAP to cross the blood-brain barrier, evidencing antitumor activity against brain metastases (Lin et al., 2008; Lin et al., 2009; Johnston et al., 2017). The LAP mechanism of action involves reversible binding to the adenosine triphosphate site, stopping cellular growth and proliferation, which results in enhanced apoptosis (Spector et al., 2005; Schroeder et al., 2014). Furthermore, LAP undergoes extensive biotransformation in humans leading to a number of metabolites, including N- and O-dealkylated products (N-LAP and O-LAP, respectively; see Figure 1) (Medina and Goodin, 2008; Towles et al., 2016).

Lapatinib gives rise to T cell-mediated hypersensitivity reactions in the skin explant assay (Ahmed et al., 2019). Moreover, dermatological adverse events including papulopustular rash, pruritus, xerosis, and nail abnormalities have been reported with LAP, similar to those described for many other TKIs (Friedman et al., 2016). Generally, hypersensitivity reactions occur when a drug (acting as a hapten) has the ability to trigger an immune response. This can be accomplished by different pathways, including noncovalent interactions of drugs with immune receptors or covalent binding of drugs to a protein, producing hapten-protein conjugates, which can induce an immune response. It is noteworthy that sometimes the drug itself does not participate in the key event but instead a reactive metabolite or a photoactivated species becomes covalently bound to the protein and generates the allergic process (Nuin et al., 2016; Limones-Herrero et al., 2020). In this context, photoallergic reactions are related to immunological mechanisms, in which the photoactivated drug (photosensitizer) is the key chemical entity capable to interact with proteins (Andreu et al., 2010). Indeed, it has recently been demonstrated that LAP and N-LAP are able to induce phototoxicity and photogenotoxicity to cells, while O-LAP did not display any photosensitized damage. Besides, the parent drug showed higher activity in membrane phototoxicity and protein oxidation than N-LAP (García-Lainez et al., 2020).

Interestingly, LAP is known to bind to blood proteins (>99%), mainly serum albumins (SAs) and α_1_-acid glycoproteins (AGs) (Medina and Goodin, 2008). The binding of drugs to transport proteins, such as SAs and AGs, attracts increasing attention because key properties such as drug function, pharmacokinetics, toxicity and transport to the target cells are strongly modulated through drug@protein complexation (Krasner, 1972; Peters, 1995). Moreover, exposure of these complexes to sunlight may result in photosensitivity disorders mainly associated with phototoxicity and photoallergy (Vargas et al., 1993; Miranda et al., 1994; Quintero and Miranda, 2000; Cosa, 2004; Montanaro et al., 2009; Nuin et al., 2016; Vayá et al., 2016; Blakely et al., 2019). In this context, it is known that drugs containing the quinazoline moiety (the core chromophore of LAP) can induce skin diseases such as allergic photodermatitis (Ishikawa et al., 1994; Selvam and Kumar, 2011).

Fluorescence and transient absorption spectroscopies are useful techniques to investigate drug/protein interactions. This is because excited state properties of drugs are very sensitive to the microenvironment; thus, the yield of transients formation, as well as their spectral profile and kinetic evolution, may be strongly affected by the surroundings of the investigated chromophore (Vayá et al., 2014). In particular, fluorescence and femtosecond transient absorption are highly sensitive techniques that may provide key information on the structural and dynamic features of drug@protein complexation, revealing the nature of the early primary processes occurring from the excited states in a time window from the fs to the ns range, such as intersystem crossing, energy/electron transfer or charge separation. In a parallel approach, molecular dynamics (MD) simulations can be used to study drug@biomolecule interactions (Spitaleri and Rocchia, 2019). Thus, properties such as the strength of interaction and the conformational orientation of a drug in the neighborhood of the amino acid residues of the protein binding sites can be investigated in detail (Pérez-Ruiz et al., 2017; Pinheiro and Curutchet, 2017; Molins-Molina et al., 2019; Spitaleri and Rocchia, 2019).

In this framework, preliminary findings on LAP@SA complexation point to a moderate binding of the drug in the so-called site III (subdomain IB) of the protein (Shen et al., 2015; Wilson et al., 2015; Kabir et al., 2016). In addition, a recent photophysical study on the interactions between LAP, N-LAP or O-LAP and HSA (Vayá et al., 2020) has shown that within the constrained environment provided by the HSA cavities emission occurs from long-lived locally excited (LE) states, whereas in the bulk solution shorter-lived (70–90 ps) intramolecular charge transfer (ICT) states predominate. This is related to the relative conformational orientation of the furan vs. the quinazoline ring in the different media.

With this background, the aim of the present work is to obtain relevant information about the complexation of LAP and its N- and O-dealkylated metabolites with serum proteins (SAs and AGs) from human and bovine origin, as a model for the first step involved in (photo)sensitivity reactions. To this end, a combination of photophysical techniques (fluorescence and femtosecond transient absorption spectroscopies) with MD simulation studies has been employed. The obtained results are relevant in connection with the capability of LAP to elicit hypersensitivity reactions with special emphasis on its photosensitizing potential.

## Experimental Section

### Chemicals and Reagents

Lapatinib (CAS 231277-92-2), serum albumins and α_1_-acid glycoproteins, from human and bovine origin, were purchased from Sigma-Aldrich (Madrid, Spain). N-De[2-(methylsulfonyl)ethyl lapatinib (N-LAP, CAS 697299-82-4) and O-De(3-fluorobenzyl) lapatinib ditosylate salt (O-LAP; CAS 1268997-70-1) were provided by Santa Cruz Biotechnology (Dallas, United States) and Toronto Research Chemicals (North York, Canada), respectively. PBS buffer was prepared by dissolving phosphate-buffered saline tablets (Sigma-Aldrich) using ultrapure water from a Millipore (Milli-Q Synthesis) system.

### Spectroscopic Measurements

Steady-state absorption spectra were recorded in a JASCO V-760 spectrophotometer. Steady-state fluorescence spectra were obtained using a JASCO-8500 spectrofluorometer system provided with a monochromator in the wavelength range 200–900 nm, with an excitation wavelength of 295 or 340 nm at 25°C. Measurements on drug or metabolite@protein complexes were performed in aerated PBS of 1:1 M ratio mixtures at 5 μM. Competing interactions were evaluated for solutions containing LAP (or its metabolites) within a mixture of proteins in a 1:1:1 M ratio (each component at 5 μM).

Time-resolved fluorescence measurements were done using an EasyLife X system containing a sample compartment composed of an automated Peltier cuvette holder to control the temperature, a pulsed LED excitation source and a lifetime detector. The employed LED excitation source was 340 nm, with emission filter of GG400.

The UV and fluorescence measurements were recorded using 10 × 10 mm^2^ quartz cells at 25°C. The absorbance of the samples at the excitation wavelength was kept below 0.1.

Femtosecond transient absorption experiments were performed using a typical pump-probe system. The femtosecond pulses were generated with a mode-locked Ti-Sapphire laser of a compact Libra HE (4 W power at 4 kHz) regenerative amplifier delivering 100 fs pulses at 800 nm (1 mJ/pulse). The output of the laser was split into two parts to generate the pump and the probe beams. Thus, tunable femtosecond pump pulses were obtained by directing the 800 nm light into an optical parametric amplifier. In the present case, the pump was set at 330 nm and passed through a chopper prior to focus onto a rotating cell (1 mm optical path) containing the samples in organic or aqueous solution. The white light used as probe was produced after part of the 800 nm light from the amplifier traveled through a computer controlled 8 ns variable optical delay line and impinge on a CaF_2_ rotating crystal. This white light was in turn split in two identical portions to generate reference and probe beams that then were focused on the rotating cell containing the sample. The pump and the probe were made to coincide to interrogate the sample. The power of the pump beam was set to 180 μW. A computer-controlled imaging spectrometer was placed after this path to measure the probe and the reference pulses to obtain the transient absorption decays/spectra. The experimental data were treated and compensated by the chirp using the ExciPro program.

### Molecular Docking

These calculations were performed using GOLD 5.8.1 program (CCDC, 2020) and the protein coordinates were taken from: 1) the crystal structure of HSA in complex with hemin and myristic acid (PDB entry 1O9X) (Zunszain et al., 2003); 2) the crystal structure of BSA in complex with 3,5-diiodosalicylic acid (PDB entry 4JK4, chain A) (Sekula et al., 2013); 3) the crystal structure of HAG in the unbound form (PDB entry 3KQ0) (Schonfeld et al., 2008); and 4) our previously reported homology model of BAG using the Phyre2 (Kelley et al., 2015) homology modeling web server (Limones-Herrero et al., 2017). The experimental procedure was similar to that described for: 1) 2-acetoxy-4-trifluoromethylbenzoic acid (triflusal) (Molins-Molina et al., 2019) and HSA protein with the exception that the position of hemin was used to define the binding pocket, and the radius was set to 10 Å; and 2) carprofen methyl ester and the homology model of BSA (Limones-Herrero et al., 2017). The protonated forms of the ligands (secondary and primary amines) were employed.

### Molecular Dynamics Simulation Studies

The highest score solution obtained by docking was subjected to 100 ns of dynamic simulation. The experimental protocol involved: 1) the minimization of the ligands (LAP, N-LAP and O-LAP); 2) the generation and minimization of the binary LAP@protein, N-LAP@protein, and O-LAP@protein complexes (protein = HSA, BSA, HAG, and BAG) using the poses obtained by docking; and 3) MD simulations of the resulting minimized ligand@protein complexes. The protocol was performed as described for triflusal and carprofen methyl ester (Limones-Herrero et al., 2017; Molins-Molina et al., 2019). The analysis of the trajectories and the rmsd of the atomic positions of the protein and the ligands during the simulation were analyzed by using the cpptraj module in AMBER 16 (Case et al., 2016). The binding free energies of LAP in the LAP@HSA and LAP@BSA complexes were calculated using the MM/PBSA (Miller et al., 2012) approach in explicit water (generalized Born, GB) as implemented in Amber. The protein figures disclosed were created by using the molecular graphics program PyMOL (DeLano, 2020). For figures related to HSA, BSA and HAG proteins, the amino acid numbering described in PDB entries 1O9X, 4JK4, and 3KQ0, respectively, was employed. For figures related to BAG protein, the numbering of the protein sequence was used.

## Results and Discussion

The photobehavior of LAP and its metabolites was investigated in aqueous buffer solution and in the presence of an equimolar amount of protein. For this purpose, two types of transport proteins were selected: serum albumins and α_1_-acid glycoproteins from human (HSA and HAG) and bovine (BSA and BAG) origin, respectively. As previously observed, LAP and N-LAP formed aggregates in PBS solution (Wilson et al., 2015; Vayá et al., 2020); however, upon interaction with proteins they were completely solubilized. The UV absorption spectra of LAP, N-LAP and O-LAP bound to HSA, BSA, HAG and BAG did not reveal significant differences (Supplementary Figure S1).

Emission of the protein-bound LAP (or metabolites) was first investigated at λ_exc_ = 295 nm, where both the protein and the drug absorb light. In PBS, the fluorescence of LAP was weak and unstructured (λ_max_ ∼ 475 nm) due to aggregation but most importantly due to emission from intramolecular charge transfer (ICT) states, which are favored in a twisted orientation between the furan and quinazoline rings due to the freedom in the degrees of movement of the drug in solution (Vayá et al., 2020). However, in the presence of protein, which provides a more constrained environment, a clear enhancement of LAP fluorescence in addition to quenching of the protein emission (λ_max_ ∼ 340 nm) was noticed (see Supplementary Figure S2). This effect was previously detected for the drug complexed to HSA, and was associated to singlet-singlet energy transfer (SSET) from HSA to LAP (Vayá et al., 2020). On the other hand, the spectra within each protein were unstructured and displayed their maxima at shorter wavelengths (*ca*. 450 nm) compared to the free drug in PBS. Quenching of the protein fluorescence (λ_max_ ∼ 340 nm) upon interaction with LAP, N-LAP and O-LAP was different for each complex (Supplementary Figures S2–S4; Supplementary Table S1). In general, higher quenching due to SSET was detected for the human proteins; besides, SAs appeared to induce greater deactivation than AGs. This can be associated to the encapsulation of the drug (or metabolite) inside the protein, which was stronger for LAP@protein compared to N-LAP and O-LAP.

**FIGURE 2:**
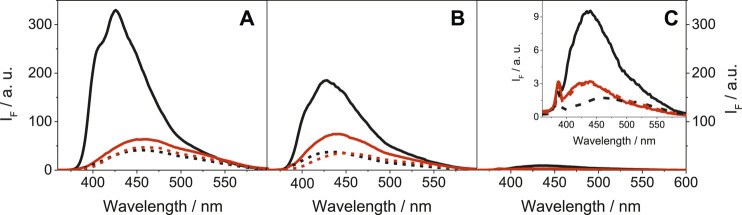
Fluorescence spectra at λ_exc_ = 340 nm for **(A)** LAP@HSA (solid black), LAP@BSA (solid red), LAP@HAG (dashed black) and LAP@BAG (dashed red); **(B)** N-LAP@HSA (solid black), N-LAP@BSA (solid red), N-LAP@HAG (dashed black) and N-LAP@BAG (dashed red); **(C)** O-LAP@HSA (solid black), O-LAP@BSA (solid red), O-LAP@HAG (dashed black) and O-LAP@BAG (dashed red). All mixtures were at 1:1 M ratio (5 μM) in PBS under air, using isoabsorptive solutions at the excitation wavelength. The inset in **(C)** shows a zoom of the low emissive states for the O-LAP@protein complexes.

More relevant from the photobiological point of view was the emission behavior of LAP and its metabolites upon excitation with UVA light (λ_exc_ = 340 nm), as this type of radiation is selectively absorbed by the drug chromophore and does not interact directly with biomolecules. The obtained results for LAP, N-LAP and O-LAP, upon excitation at 340 nm are shown in Figure 2.

In contrast to the strong and structured fluorescence with maxima at *ca*. 425 nm detected for LAP and N-LAP within HSA (Figures 2A,B), the emissions of the other ligand@protein complexes were much weaker and unstructured, and the maxima were found at longer wavelengths (∼450 nm). The behavior within HSA was associated to the more constrained environment provided by the protein but also to the frozen coplanar orientation adopted by the furan and quinazoline rings of LAP or N-LAP, which favors emission from LE (locally excited) states (Vayá et al., 2020). On the contrary, binding to BSA, HAG or BAG might disfavor emission from LE states due to a certain degree of freedom for twisting between both rings, resulting in a diminished and red-shifted emission of the encapsulated LAP or N-LAP. This would imply that the confinement and the conformational arrangement of the ligands within the protein cavities are key factors controlling the photobehavior. As regards the fluorescence lifetimes, decay kinetics were measured upon excitation at 340 nm (Supplementary Figure S5). In general, shorter lifetimes (<1 ns) were determined for LAP and N-LAP bound to AGs than those determined within SAs, which ranged from 1 to 1.5 ns (Supplementary Table S2). Besides, the decay traces were faster for N-LAP than for the parent drug, which is in line with the steady-state results. Again, little if any emission was detected for O-LAP in the presence of the different proteins, suggesting the feasibility of alternative deactivation pathways, for instance excited state deprotonation of the phenol moiety.

Furthermore, both SAs and AGs are transport proteins present in the plasma. Under normal conditions, the concentration of the former is higher; however, this situation may change significantly under a variety of conditions (for instance, during inflammatory processes), where AGs can play a significant role (Kremer et al., 1988; Bteich, 2019). Thus, it makes sense to investigate competing interactions of LAP or N-LAP with SAs and AGs in protein mixtures, upon selective excitation of the drug chromophore, to check the relative affinities to both types of proteins.

**FIGURE 3:**
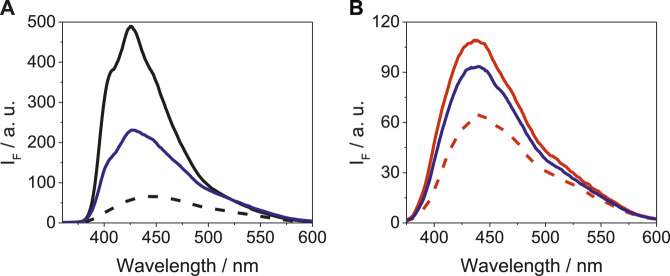
Fluorescence spectra at λ_exc_ = 340 nm for **(A)** LAP@HSA (solid black), LAP@HAG (dashed black) and LAP@HSA+HAG (solid blue), and **(B)** LAP@BSA (solid red), LAP@BAG (dashed red) and LAP@BSA+BAG (solid blue). For LAP@protein, solutions of 1:1 M ratio (5 μM) were prepared, while for LAP@protein1+protein2, solutions of 1:1:1 M ratio (5 μM) were used.

As it is shown in Figure 3, the emission spectra of LAP@HSA+HAG upon excitation at 340 nm was intermediate between the LAP@HSA and the LAP@HAG profiles; however, the shape and position of the band was more similar to that of LAP@HSA. A similar trend was also detected for LAP in a mixture of BSA and BAG, where LAP preferentially binds to BSA. Competing interactions of LAP in mixtures containing other protein combinations are shown in Supplementary Figure S6. Thus, it can be concluded that LAP interacts more strongly with the human proteins than with those of bovine origin; the weakest interaction was evidenced for BAG. A similar behavior was revealed for N-LAP in a mixture of proteins, as it can be deduced from the emission spectra shown in Supplementary Figure S7.

**FIGURE 4:**
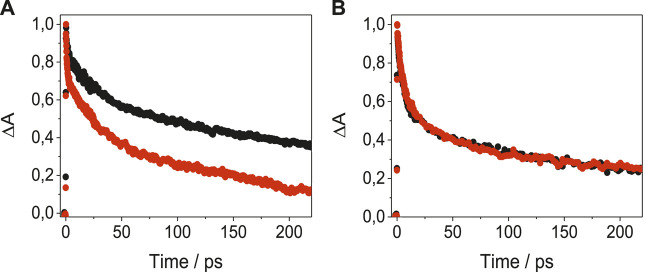
Femtosecond transient absorption decays for **(A)** LAP@HSA (black) and LAP@BSA (red) monitored at 530 nm, and for **(B)** LAP@HAG (black) and LAP@BAG (red) monitored at 550 nm, in PBS after excitation at 330 nm.

In order to get a deeper insight into the nature of LAP-protein binding, the photobehavior of the complexes was examined in the very early stages after exposure to UVA light, by means of femtosecond transient absorption measurements, upon excitation at 330 nm. The spectral shapes of the species detected for LAP in its complexes with BSA, HAG and BAG (Supplementary Figure S8) were in good accordance with that obtained previously for LAP@HSA (Vayá et al., 2020). Thus, a transient absorption band with relative maxima at *ca*. 425 and 530 nm was observed for LAP@BSA; similar profiles were noticed for LAP@AGs, with maxima around 425 and 550 nm. These transients were assigned to the LE singlet-singlet absorption species; their kinetic traces decayed double-exponentially (Figure 4). Thus, for SAs, short components with lifetimes of *ca*. 12 ps were evidenced, while the longer components were species-dependent and decayed faster for BSA than for HSA, reaching the ns time-scale. For the AGs, the short components decayed with lifetimes around 6 ps while the long ones survived until the ns time-scale. The two lifetime components could be associated to the LE states of LAP within the constrained environment provided by the protein; the short components would arise from the reorganization of the drug in the initial steps after excitation, whereas the long ones would correspond to the stabilized conformation within the protein cavities. Actually, the signature of ICT states was not evidenced in any of the LAP@protein complexes.

In order to understand the marked experimental differences in the photobehavior of LAP and its metabolites interacting with SAs and AGs, the binding mode was investigated by computational studies. This was first analyzed by molecular docking using the program GOLD, version 5.8.1, and further validated by MD simulation studies.

For SAs, the ligands LAP, N-LAP and O-LAP were docked to subdomain IB. Experimental evidence to assign subdomain IB as the preferred binding site in HSA was previously obtained using selective site I and site II probes such as warfarin (WRF) and ibuprofen (IBP), respectively (Vayá et al., 2020). The resulting ligand@protein binary complexes were submerged in a truncated octahedron of water molecules and further analyzed by MD simulations (100 ns) using the molecular mechanics force field AMBER to achieve reliable models (Götz et al., 2012; Le Grand et al., 2013; Salomon-Ferrer et al., 2013). All ligand@protein complexes proved to be very stable during simulation, as evidenced by the low values of the root-mean-square deviation (rmsd) for the whole protein backbone (C^α^, C, N and O atoms) calculated for all complexes (average values range from 0.7 to 2.0 Å) (Supplementary Figures S9–S12).

**FIGURE 5:**
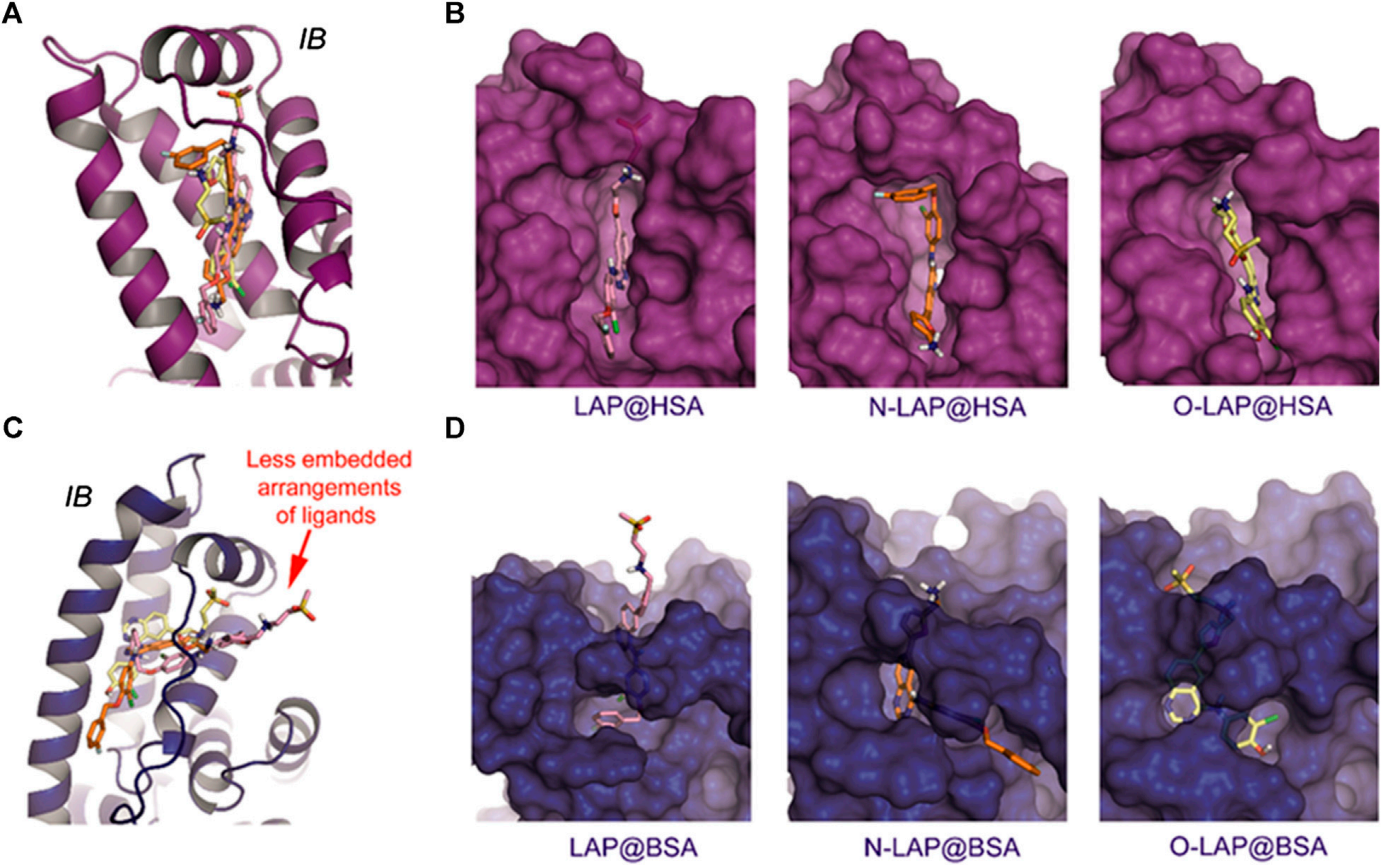
Overall view of the proposed binding modes of LAP (pink), N-LAP (orange) and O-LAP (yellow) to HSA (magenta, **A,B**) and BSA (blue, **C,D**) as obtained by MD simulation studies. **(A,C)** Superposition of the binding modes of the three ligands to subdomain IB of HSA **(A)** and BSA **(C)**. **(B,D)** Detailed views of the proposed binary ligand@HSA **(B)**, and ligand@BSA **(D)** complexes.

**FIGURE 6:**
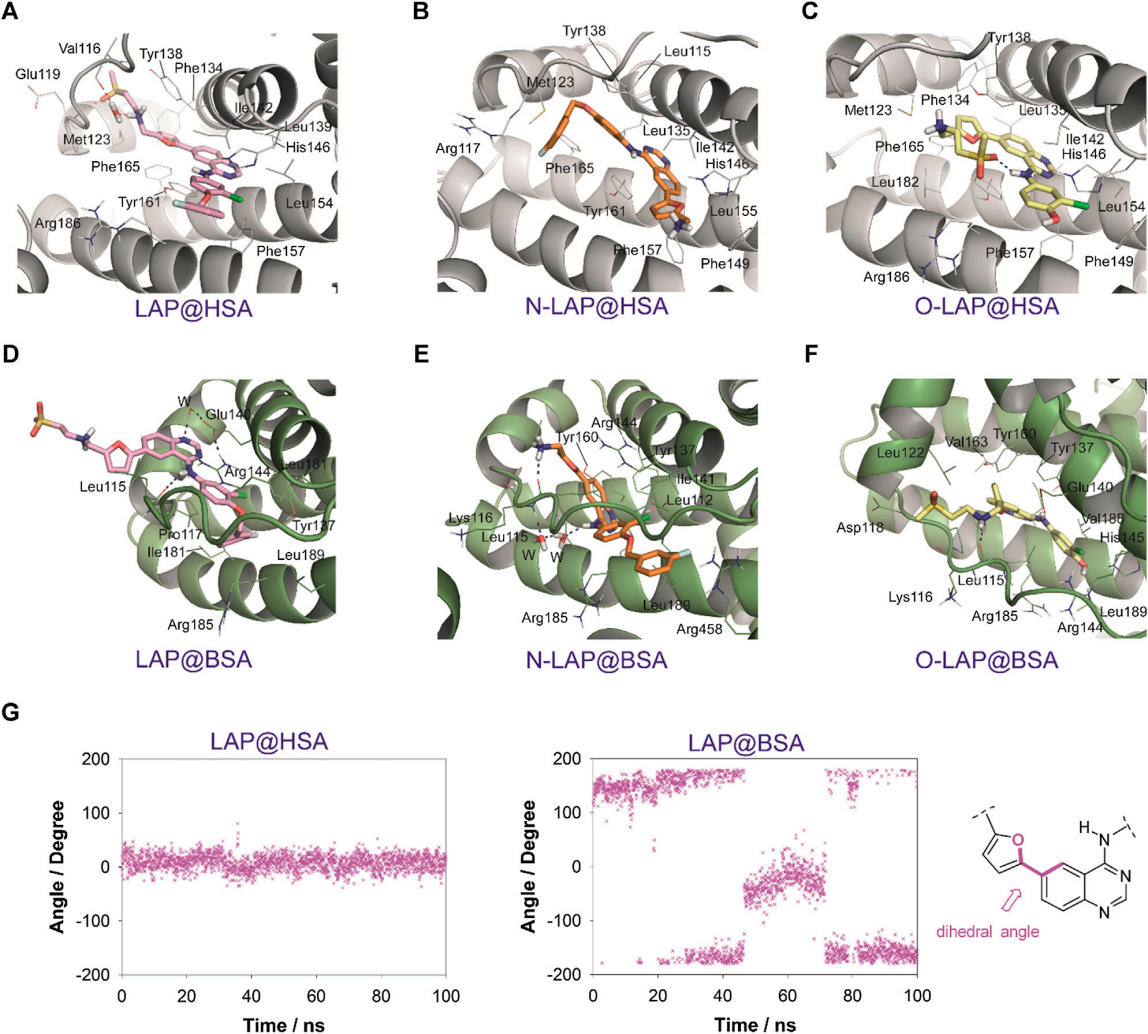
Interactions of LAP (pink, **A,D**), N-LAP (orange, **B,E**), and O-LAP (yellow, **C,F**) to HSA (gray, **A–C**) and BSA (green, **D–F**) as obtained by MD simulation studies. Snapshots after 80 ns are shown. Relevant side chain residues are shown (lines) and labeled. Ligands are indicated as sticks. Hydrogen bonding interactions between the ligands and the protein are shown as blue dashed lines. **(G)** Variation of the dihedral angle between the furyl and quinazoline rings for LAP bound to HSA or BSA during the whole simulation. The bonds involved in the dihedral angle are highlighted in pink.

The results of the MD simulation studies carried out for the ligand@SA complexes revealed that: 1) all ligands displayed a more deeply embedded arrangement in subdomain IB when bound to HSA than to BSA; 2) for the BSA complexes, a portion of the ligands was exposed to the solvent environment, particularly in the case of LAP. Specifically, the overall arrangement adopted by LAP or N-LAP was found to be clearly different in HSA compared to BSA (Figure 5). Thus, for LAP@BSA only part of the ligand interacted within the protein binding site, while N-LAP was rotated 180° within BSA to locate the ammonium moiety in close contact with the solvent. In any case, all ligands would be stabilized by numerous apolar interactions with the side chain residues within subdomain IB, as well as hydrogen bonding interactions through the ammonium group and the nitrogen atoms of the quinazoline ring. In addition, conformational analysis of the ligands within subdomain IB showed that: 1) only LAP and O-LAP would achieve an almost coplanar arrangement of the furyl and quinazoline rings upon binding to HSA, showing average dihedral angles of 8.3° and 7.3°, respectively, being their relative conformations frozen within the pocket; 2) on the contrary, free rotation around the linkage of the furyl and quinazoline rings was possible for LAP@BSA, O-LAP@BSA and the two N-LAP@SA complexes (Figure 6; Supplementary Figure S13).

**FIGURE 7:**
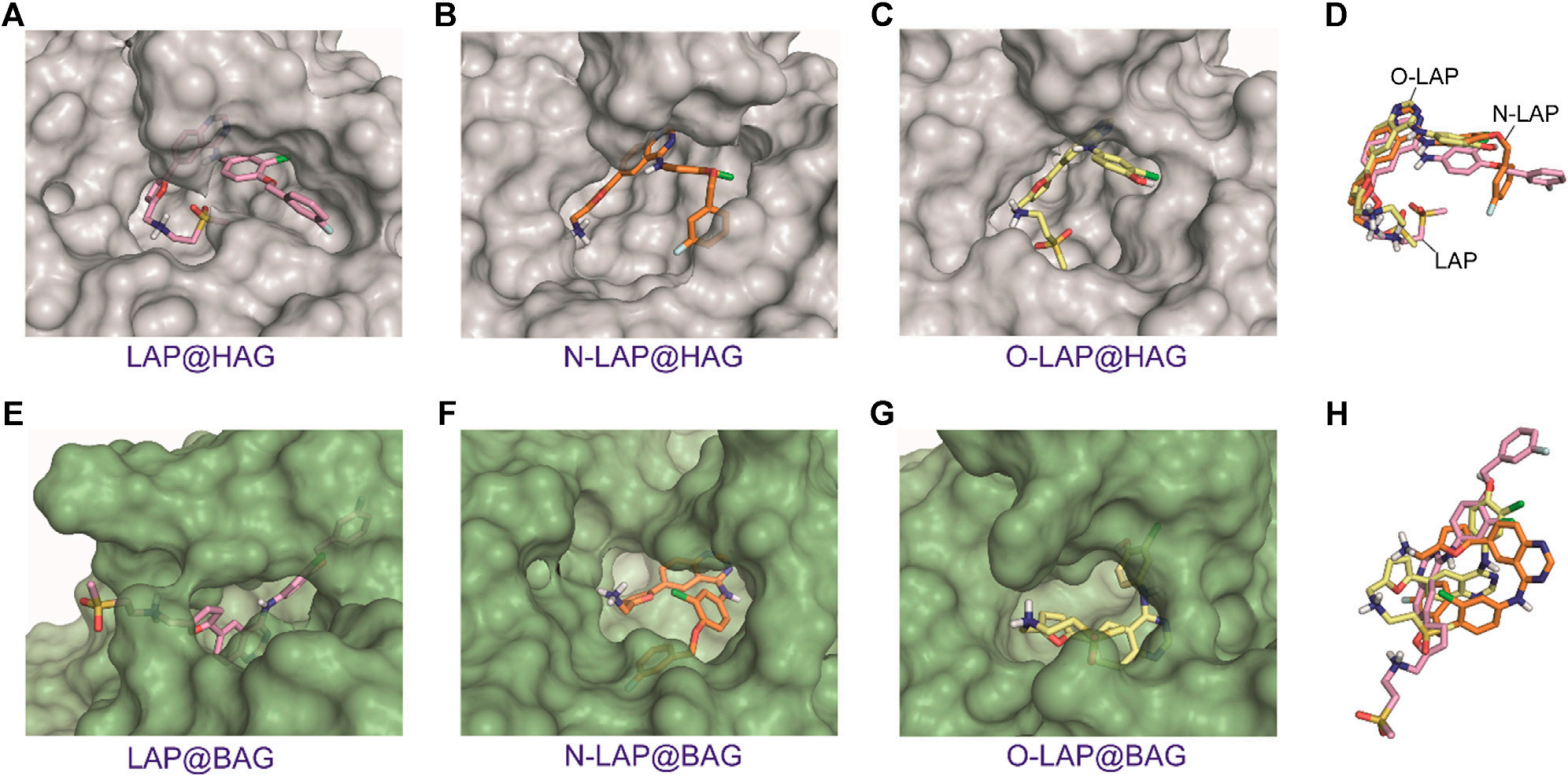
Overall view of the proposed binding modes of LAP (pink, **A,E**), N-LAP (orange, **B,F**) and O-LAP (yellow, **C,G**) to HAG (gray) and BAG (green) as obtained by MD simulation studies. **(D,H)** Superposition of the binding modes of the tree ligands in the recognition pocket of HAG **(D)** and BAG **(H)**. Note how the furyl and quinazoline rings of the ligands for the HAG complexes would be almost coplanar and all ligands would show a similar arrangement.

Unlike SAs that can undergo large conformational changes upon binding a variety of ligands, specifically in domains I and III (Curry et al., 1998; Pérez-Ruiz et al., 2017), the plasticity of AGs is intrinsically more limited. Their central motif, which is composed by an eight-stranded β-barrel and is flanked by an α-helix, makes them constrained receptacles for ligand recognition. It is therefore not surprising that the MD simulation studies carried out for the ligand@AG complexes showed that the overall flexibility of LAP or its metabolites within the binding pockets would be restricted to some extent (Figures 7A–C; Supplementary Figure S14). This effect was found to be more pronounced for the ligand@HAG complexes, where the drug and its metabolites would be well surrounded by the protein. In addition, a similar arrangement of LAP, N-LAP and O-LAP within HAG was observed, with an almost coplanar arrangement of the furyl and quinazoline rings (Figure 7D; Supplementary Figure S15). However, although this effect would favor emission from LE states, the weaker intensity observed for HAG compared to HSA could be associated with the larger binding site of the former (Supplementary Figure S16), which may reduce the matrix effect provided by the protein cavities. On the contrary, for ligand@BAG it can be summarized that: 1) no common pattern of recognition was identified; 2) more solvent-exposed complexes were obtained; and 3) no coplanarity between the aromatic moieties was observed (Figures 7E–H; Supplementary Figures S14 and S15). In general, the MD simulation results are in line with the experimental data, as they justify the higher emission of LAP bound to HSA compared with the other proteins, in addition to the stronger affinity to the human proteins, as a result of the deeper embedded arrangement with higher restriction in the degrees of movement compared to the bovine proteins. Besides, the stronger emission of N-LAP within HSA and the weaker emission of the drug and its metabolites in BAG are also explained.

Finally, the binding free energies of LAP, N-LAP and O-LAP interacting with the investigated proteins (HSA, BSA, HAG and BAG) in the corresponding ligand@protein complex were calculated using the MM/PBSA approach (Miller et al., 2012) in explicit water (generalized Born, GB) as implemented in Amber (Table 1). These energies were estimated by subtracting the free energy of the corresponding unbound components, i.e. ligand and protein, to the free energy of the ligand@protein complex. The results revealed that the binding affinity of LAP to the human proteins is higher than to the bovine ones, especially in the case of SAs. Thus, LAP would have a 1.6-fold higher affinity for HSA than for BSA. This is mainly caused by the distinct amino acid sequence of the subdomain IB for both SAs (Supplementary Figure S17). These differences in the intraprotein microenvironments that surround the ligands are responsible for the type and strength of the stabilization interactions, as it can be observed from Figures 6A–F. This effect is even greater when comparing SAs and AGs in which the ligand-binding pockets are markedly different, either in amino acid sequence (Supplementary Figure S18) as well as in their tridimensional arrangement. In addition, the affinity of both metabolites to the investigated proteins was found to be weaker than that of the parent drug, following the order LAP >> N-LAP > O-LAP. Again, these results are qualitatively in line with those obtained from the fluorescence measurements, where higher affinity of either LAP or its metabolites was found for the human proteins, with the highest one observed for LAP@HSA.

**TABLE 1:** Calculated binding free energies using MM/PBSA.^a^

Ligand	Protein	Complex	Energy
LAP	HSA	LAP@HSA	−56.7 ± 0.2^b^
—	BSA	LAP@BSA	−36.2 ± 0.3^b^
—	HAG	LAP@HAG	−54.3 ± 0.3^b^
—	BAG	LAP@BAG	−50.4 ± 0.4^b^
N-LAP	HSA	N-LAP@HSA	−43.5 ± 0.2^b^
—	BSA	N-LAP@BSA	−35.3 ± 0.2^b^
—	HAG	N-LAP@HAG	−39.1 ± 0.2^b^
—	BAG	N-LAP@BAG	−40.2 ± 0.3^b^
O-LAP	HSA	O-LAP@HSA	−37.2 ± 0.2^b^
—	BSA	O-LAP@BSA	−35.9 ± 0.2^b^
—	HAG	O-LAP@HAG	−41.3 ± 0.2^b^
—	BAG	O-LAP@BAG	−34.0 ± 0.2^b^

a Energy units = kcal mol^−1^.

b Standard error of mean.

## Conclusion

The interaction of Lapatinib (LAP) and its N- and O-dealkylated metabolites (N-LAP and O-LAP) with model proteins (serum albumins and α_1_-acid glycoproteins, SAs and AGs), of human and bovine origin, has been investigated by a combined photophysical and computational approach. In this context, the present work reveals key information on drug@protein complexation, the first step involved in a number of hypersensitivity reactions, including photosensitivity disorders. Photophysical results (fluorescence and ultrafast transient absorption) agree with strong and specific interactions of the drug and its metabolites with the selected proteins, pointing to a higher affinity to the human proteins, especially in the case of the LAP@HSA complex. The observed behavior can be rationalized by the coplanar orientation adopted by the furan and quinazoline rings within subdomain IB of HSA, in conjunction with the degree of confinement provided by this constrained intraprotein microenvironment. The experimental findings can be undoubtedly explained by the MD simulation studies. Thus, the weaker emission observed within BSA is explained by the higher exposure of part of the ligand to the bulk solution and also by the conformational arrangements and the degrees of freedom within the BSA binding site. Finally, the calculated binding energies for ligand@protein complexes are also in line with the relative affinities found in the competition experiments.

## Data Availability Statement

The raw data supporting the conclusions of this manuscript will be made available by the authors, without undue reservation, to any qualified researcher.

## Author Contributions

All authors have realized substantial, direct, and intellectual contribution to the work, and approved it for publication.

## Funding

Financial support from the Spanish Government (RYC-2015-17737, CTQ2017-89416-R, SAF2016-75638-R ISCIII grants RETICS ARADyAL (RD16/0006/0004 and RD16/0006/0001), PI16/01877 and CPII16/00052), Consellería d’Educació Cultura i Esport (PROMETEO/2017/075), the Xunta de Galicia [ED431B 2018/04 and Centro singular de investigación de Galicia accreditation 2019-2022 (ED431G 2019/03)] and the European Regional Development Fund is gratefully acknowledged.

## Conflict of Interest

The authors declare that the research was conducted in the absence of any commercial or financial relationships that could be construed as a potential conflict of interest.

The reviewer DM declared a shared affiliation, with no collaboration, with one of the authors, CM, to the handling editor at the time of review.
