# Supplementary material for: Protein Binding of Lapatinib and Its N- and O-Dealkylated Metabolites Interrogated by Fluorescence, Ultrafast Spectroscopy and Molecular Dynamics Simulations
Source: Front Pharmacol. 2020 Oct 30;11:576495. doi: 10.3389/fphar.2020.576495 (PMC7662899; doi:10.3389/fphar.2020.576495)
Supplement: Supplementary file 1 [file DataSheet1_v1.pdf]

Supplementary Material

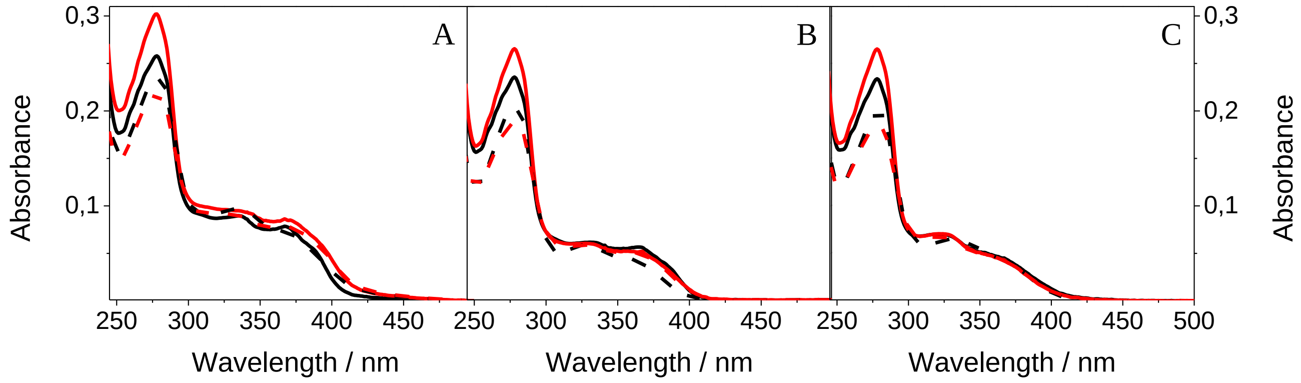

FIGURE S1. UV absorption spectra of LAP (A), N-LAP (B) and O-LAP (C) in the presence of equimolar mixtures (5 μM) of HSA (black), BSA (red), HAG (dashed black) and BAG (dashed red) in PBS.

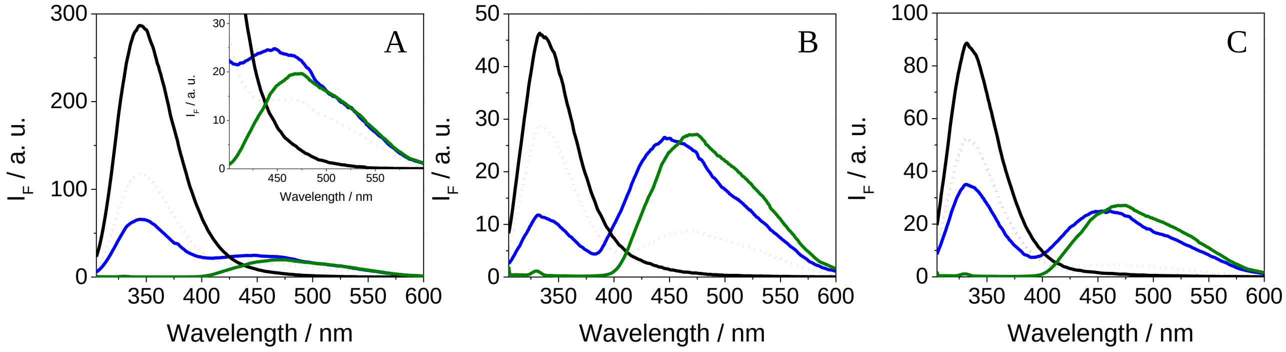

FIGURE S2. Fluorescence spectra of protein (black), LAP (green) and LAP@protein at 1:1 molar ratio (solid blue) in PBS under air, using isoabsorptive solutions at λ_{exc} = 295 nm for (A) BSA, (B) HAG and (C) BAG. The simulated emission of LAP@protein, determined as explained elsewhere (Vayá et al., 2013) taking into account the percentage of photons absorbed by the protein and LAP at 295 nm, and considering no interaction between the two entities, is shown in dotted blue. The inset in (A) shows a zoom of the emission profiles between 400 and 600 nm.

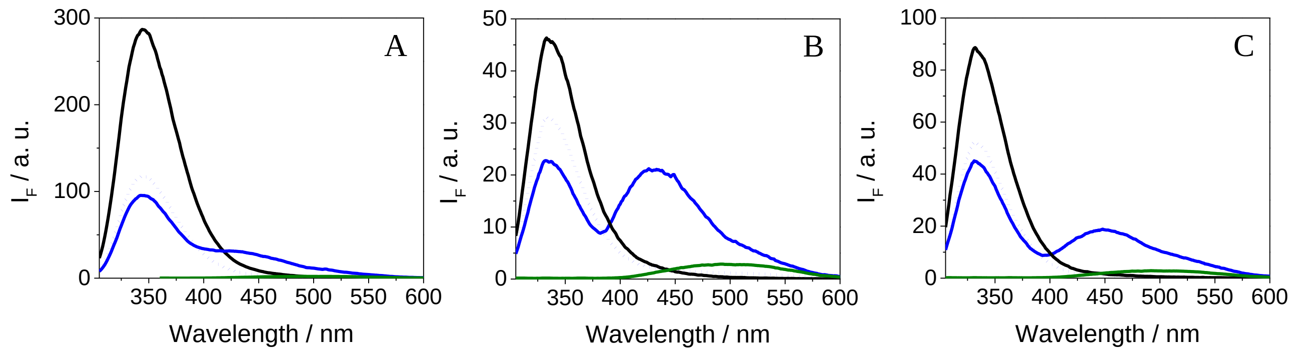

FIGURE S3. Fluorescence spectra of protein (black), N-LAP (green) and N-LAP@protein at 1:1 molar ratio (solid blue) in PBS under air, using isoabsorptive solutions at λ_{exc} = 295 nm for (A) BSA, (B) HAG and (C) BAG. The simulated emission of N-LAP@protein, determined as explained elsewhere (Vayá et al., 2013) taking into account the percentage of photons absorbed by the protein and N-LAP at 295 nm, and considering no interaction between the two entities, is shown in dotted blue.

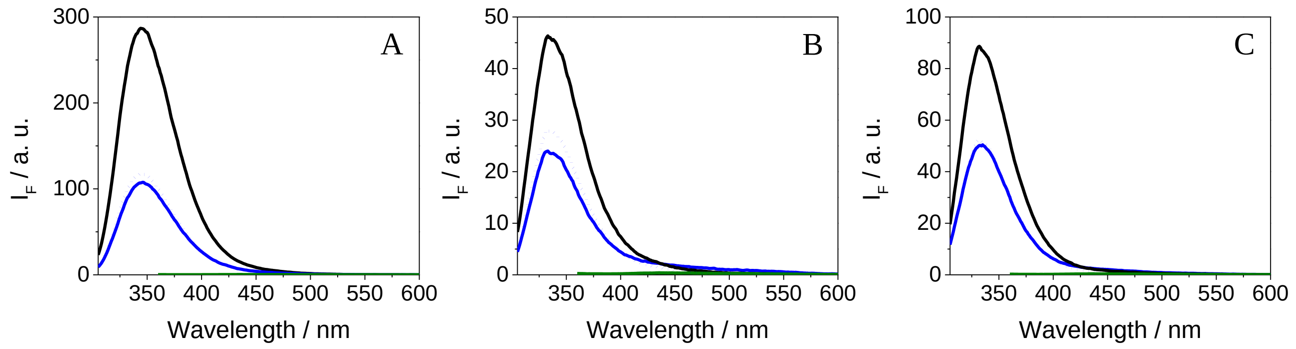

FIGURE S4. Fluorescence spectra of protein (black), O-LAP (green) and O-LAP@protein at 1:1 molar ratio (solid blue) in PBS under air, using isoabsorptive solutions at λ_{exc} = 295 nm for (A) BSA, (B) HAG and (C) BAG. The simulated emission of O-LAP@protein, determined as explained elsewhere (Vayá et al., 2013) taking into account the percentage of photons absorbed by the protein and O-LAP at 295 nm, and considering no interaction between the two entities, is shown in dotted blue.

TABLE S1. Percentage of the protein fluorescence decrease at 340 nm in the drug or metabolite@protein complexes upon excitation at 295 nm.

	HSA[a]	BSA	HAG	BAG
**LAP**	54	44	60	33
**N-LAP**	37	19	26	15
**O-LAP**	13	9	15	0

[a] Results from (Vayá et al., 2020).

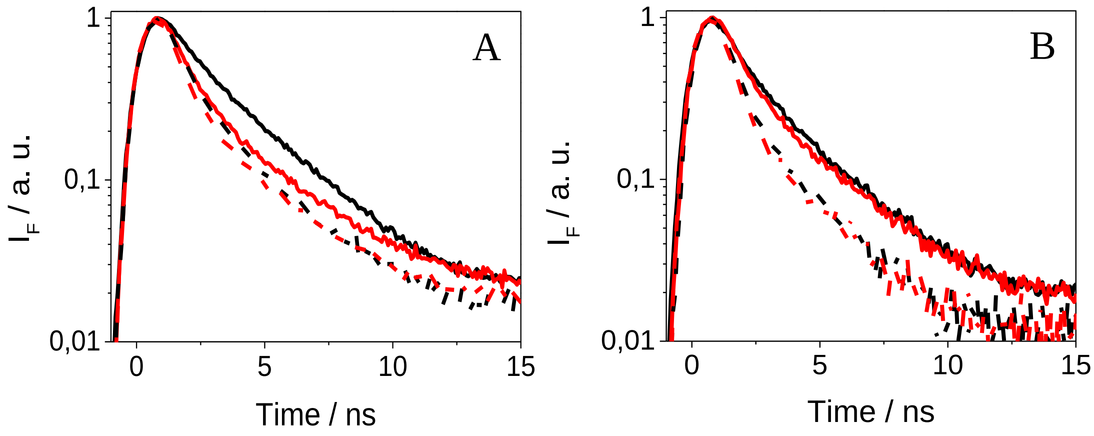

FIGURE S5. Fluorescence decay traces at λ_{exc} = 340 nm for (A) LAP@HSA (solid black), LAP@BSA (solid red), LAP@HAG (dashed black) and LAP@BAG (dashed red) and (B) N-LAP@HSA (solid black), N-LAP@BSA (solid red), N-LAP@HAG (dashed black) and N-LAP@BAG (dashed red). All mixtures were at 1:1 molar ratio (5 μM) in PBS under air, using isoabsorptive solutions at the excitation wavelength.

TABLE S2. Fluorescence lifetimes for drug/or metabolite@protein complexes upon excitation at 340 nm.

τ_F / ns	HSA[a]	BSA	HAG	BAG
**LAP**	1.5	1.0	0.9	0.8
**N-LAP**	1.2	1.1	0.5	0.4
**O-LAP**[b]	N/A	N/A	N/A	N/A

[a] Results from ref. (Vayá et al., 2020). [b] Lifetimes for the O-LAP@protein complexes could not be measured due to their very low fluorescence yields.

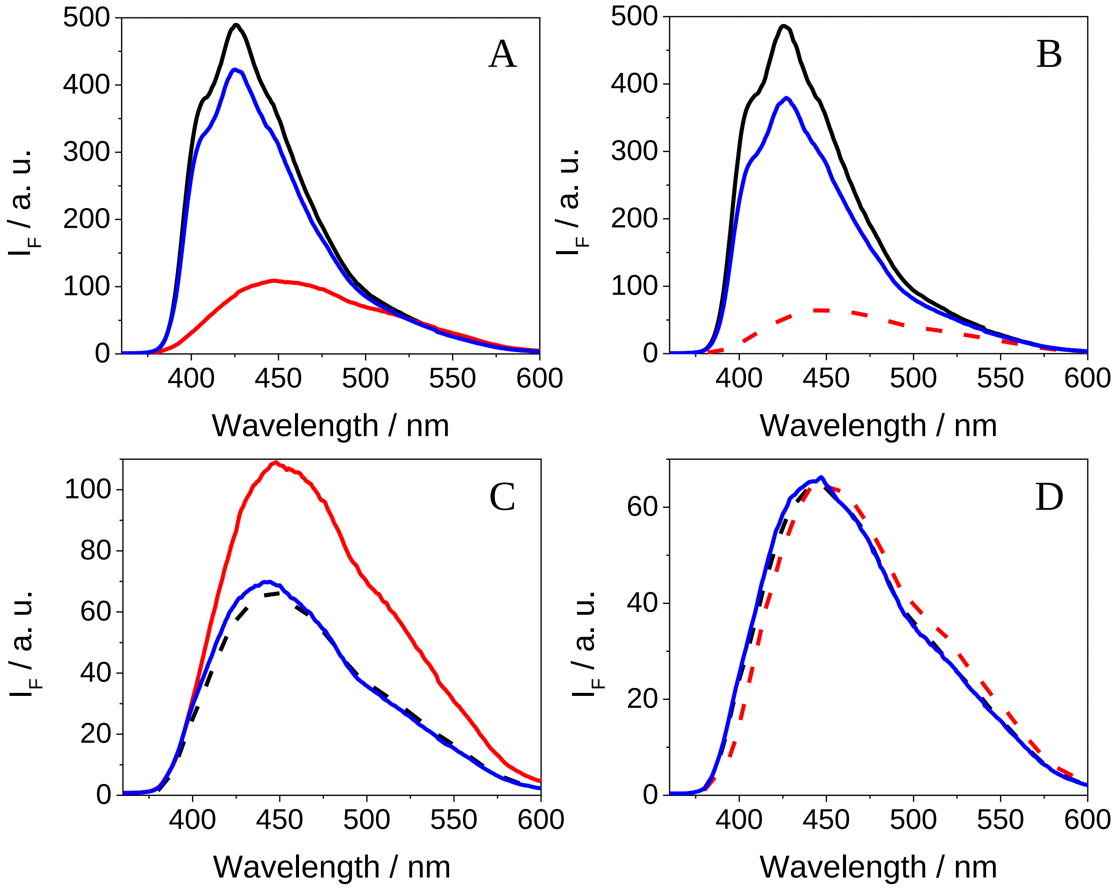

FIGURE S6. Fluorescence spectra at λ_{exc} = 340 nm for (A) LAP@HSA (solid black), LAP@BSA (solid red) and LAP@HSA+BSA (solid blue); (B) LAP@HSA (solid black), LAP@BAG (dashed red) and LAP@HSA+BAG (solid blue); (C) LAP@BSA (solid red), LAP@HAG (dashed black) and LAP@BSA+HAG (solid blue); (D) LAP@HAG (dashed black), LAP@BAG (dashed red) and LAP@HAG+BAG (solid blue). For LAP@protein, solutions of 1:1 molar ratio (5 μM) were prepared, while for LAP@protein1+protein2, solutions of 1:1:1 molar ratio (5 μM) were used.

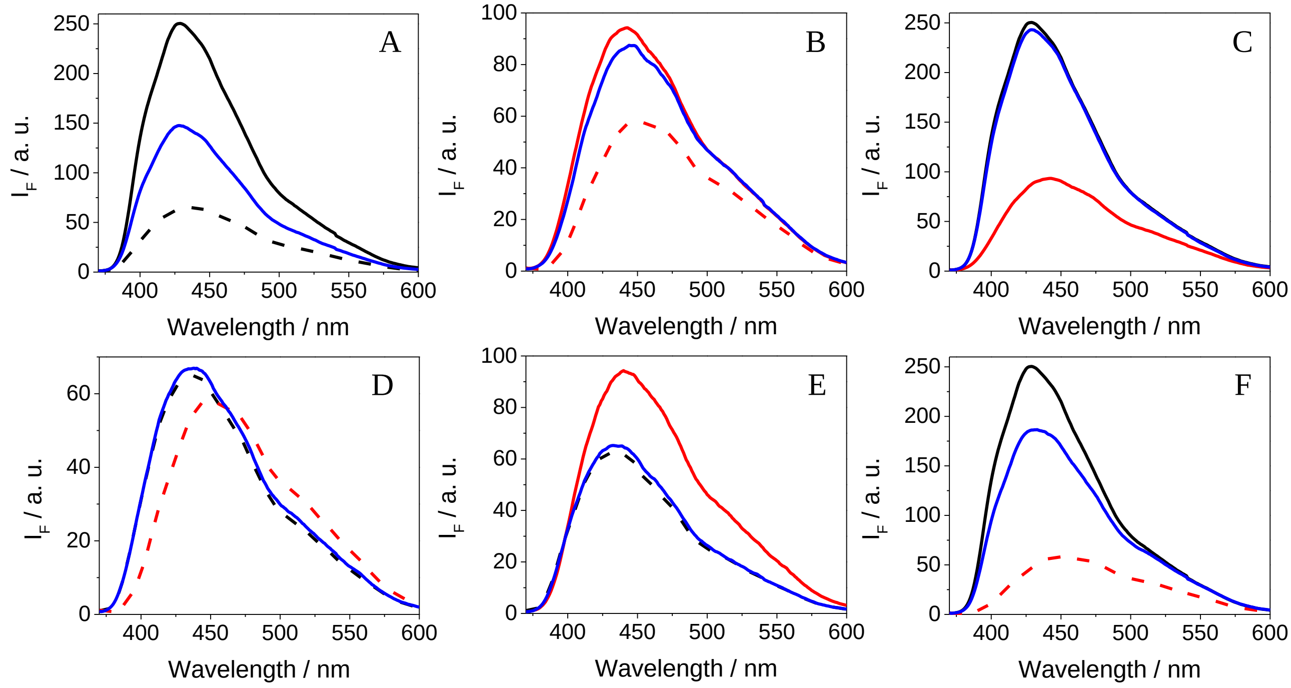

FIGURE S7. Fluorescence spectra at λ_{exc} = 340 nm for (A) N-LAP@HSA (solid black), N-LAP@HAG (dashed black) and N-LAP@HSA+HAG (solid blue); (B) N-LAP@BSA (solid red), N-LAP@BAG (dashed red) and N-LAP@BSA+BAG (solid blue); (C) N-LAP@HSA (solid black), N-LAP@BSA (solid red) and N-LAP@HSA+BSA (solid blue); (D) N-LAP@HAG (dashed black), N-LAP@BAG (dashed red) and N-LAP@HAG+BAG (solid blue); (E) N-LAP@BSA (solid red), N-LAP@HAG (dashed black) and N-LAP@BSA+HAG (solid blue); and (F) N-LAP@HSA (solid black), N-LAP@BAG (dashed red) and N-LAP@HSA+BAG (solid blue). For N-LAP@protein, solutions of 1:1 molar ratio (5 μM) were prepared, while for N-LAP@protein1+protein2, solutions of 1:1:1 molar ratio (5 μM) were used.

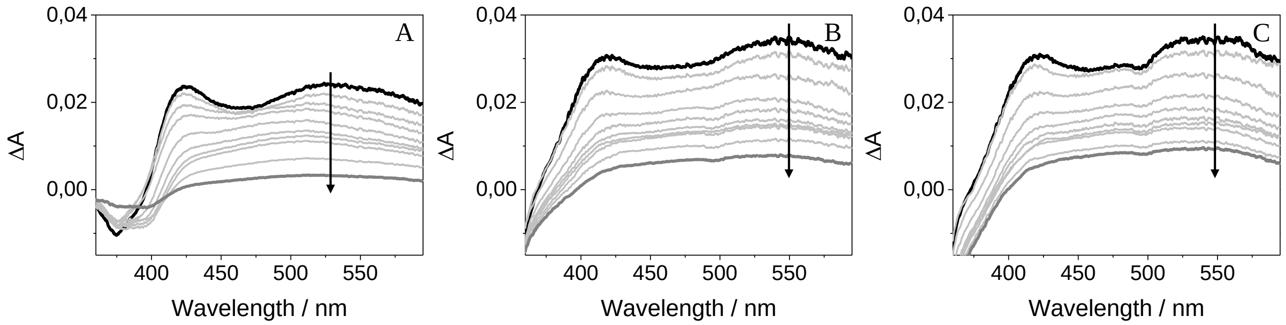

FIGURE S8. Femtosecond transient absorption spectra from 1 to 200 ps for (A) LAP@BSA, (B) LAP@HAG and (C) LAP@BAG at 1:1 molar ratio (3×10^{-4} M) in PBS at λ_{exc} = 330 nm.

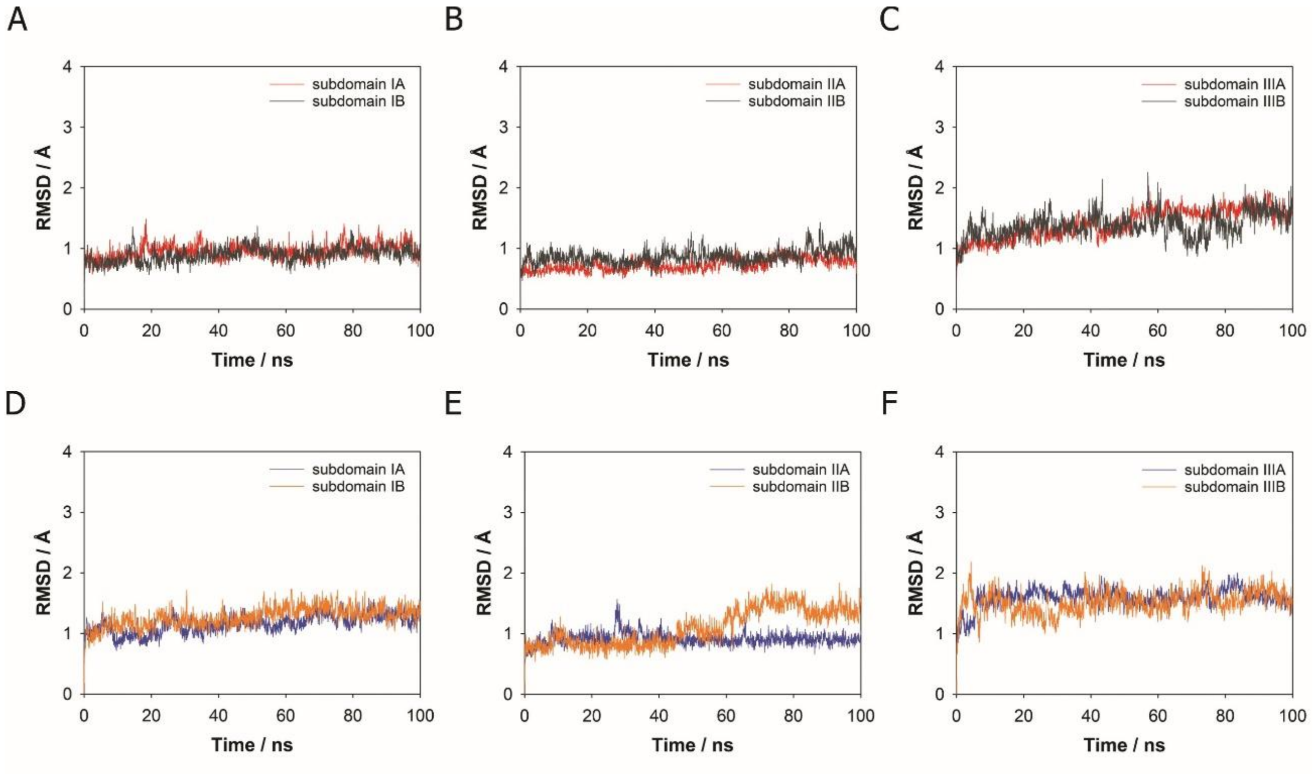

FIGURE S9. RMSD plots for the protein backbone (C^{α}, C, N, and O atoms) calculated in the complexes LAP@BSA (A-C), and LAP@HSA (D-F) obtained from MD simulations studies. The rmsd values of the six subdomains of the protein are shown. Note how the rmsd values are low. The average values range from 0.9 Å to 1.6 Å (LAP@HSA) and 0.9 Å to 1.4 Å (LAP@BSA).

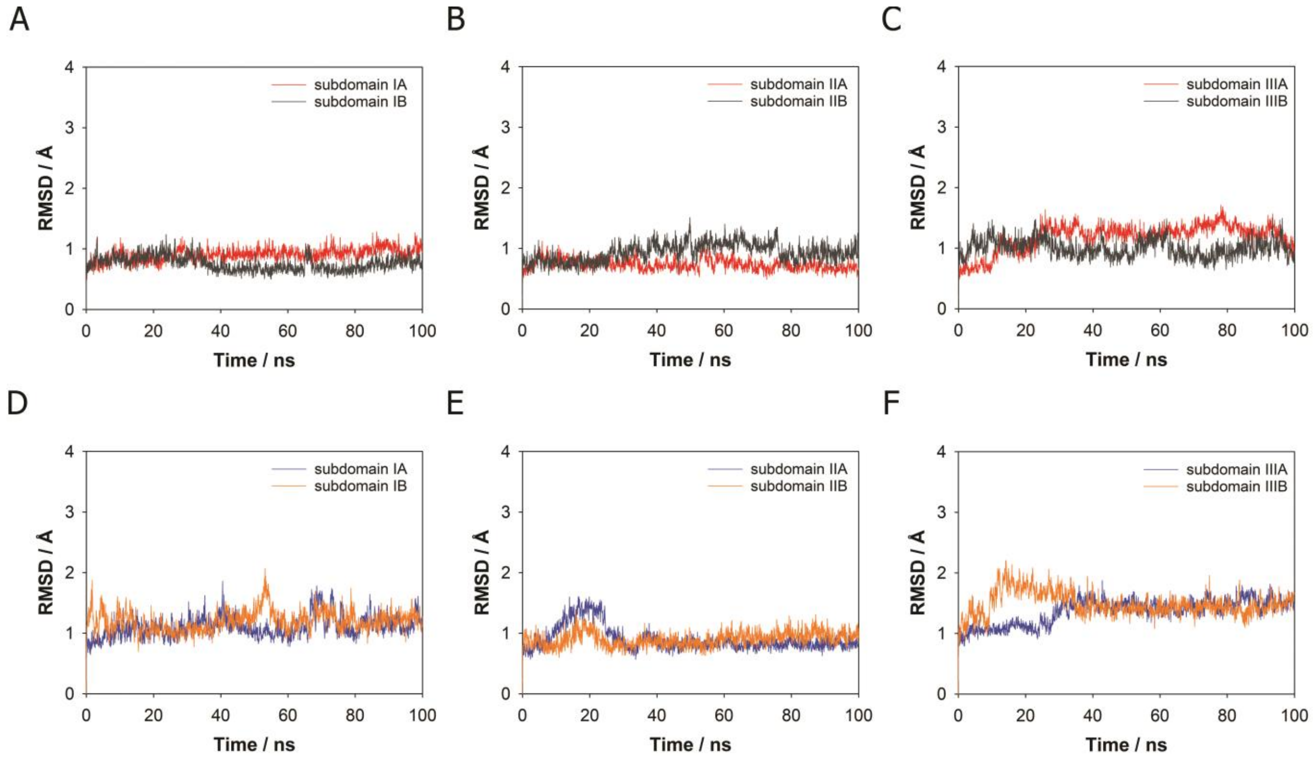

FIGURE S10. RMSD plots for the protein backbone (C^{α}, C, N, and O atoms) calculated in the complexes N-LAP@BSA (A-C), and N-LAP@HSA (D-F) obtained from MD simulations studies. The rmsd values of the six subdomains of the protein are shown. Note how the rmsd values are low. The average values range from 0.9 Å to 1.5 Å (N-LAP@HSA) and 0.7 Å to 1.2 Å (N-LAP@BSA).

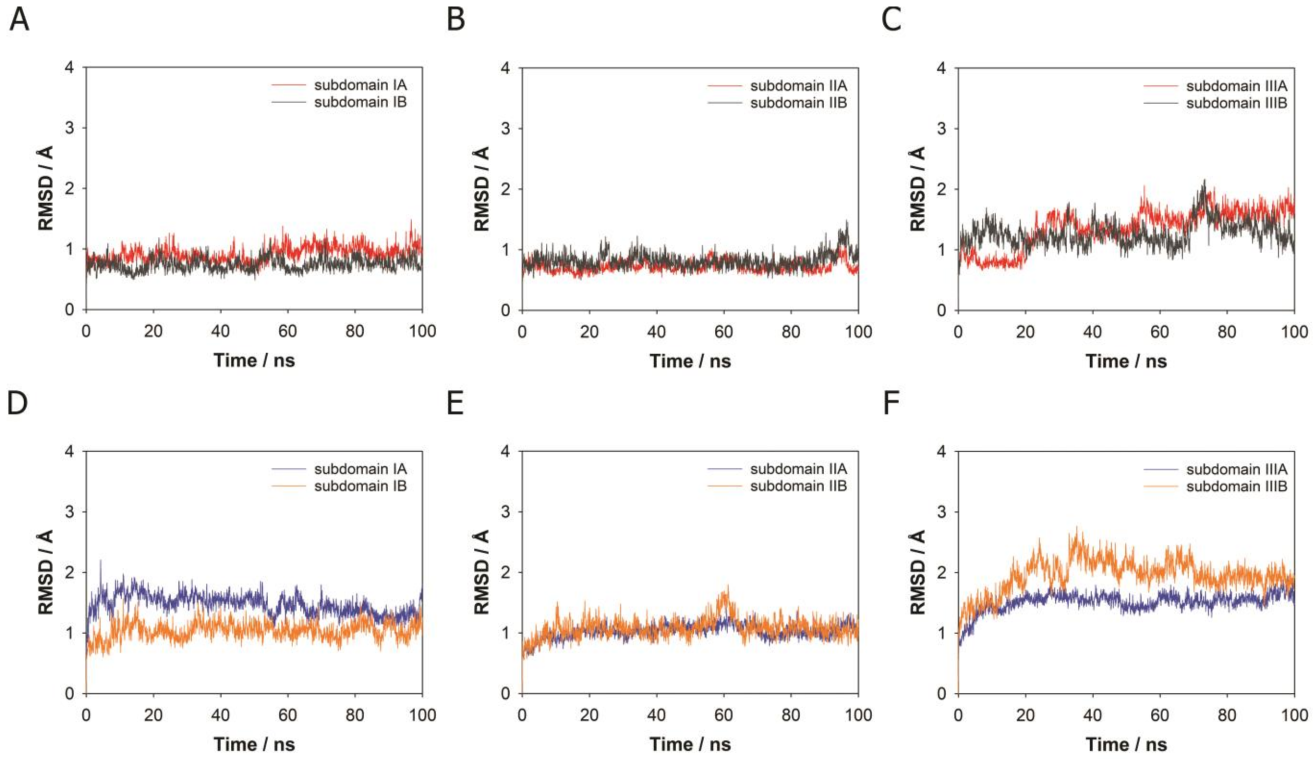

FIGURE S11. RMSD plots for the protein backbone (C^{α}, C, N, and O atoms) calculated in the complexes O-LAP@BSA (A-C), and O-LAP@HSA (D-F) obtained from MD simulations studies. The rmsd values of the six subdomains of the protein are shown. Note how the rmsd values are low. The average values range from 1.0 Å to 2.0 Å (O-LAP@HSA) and 0.7 Å to 1.4 Å (O-LAP@BSA).

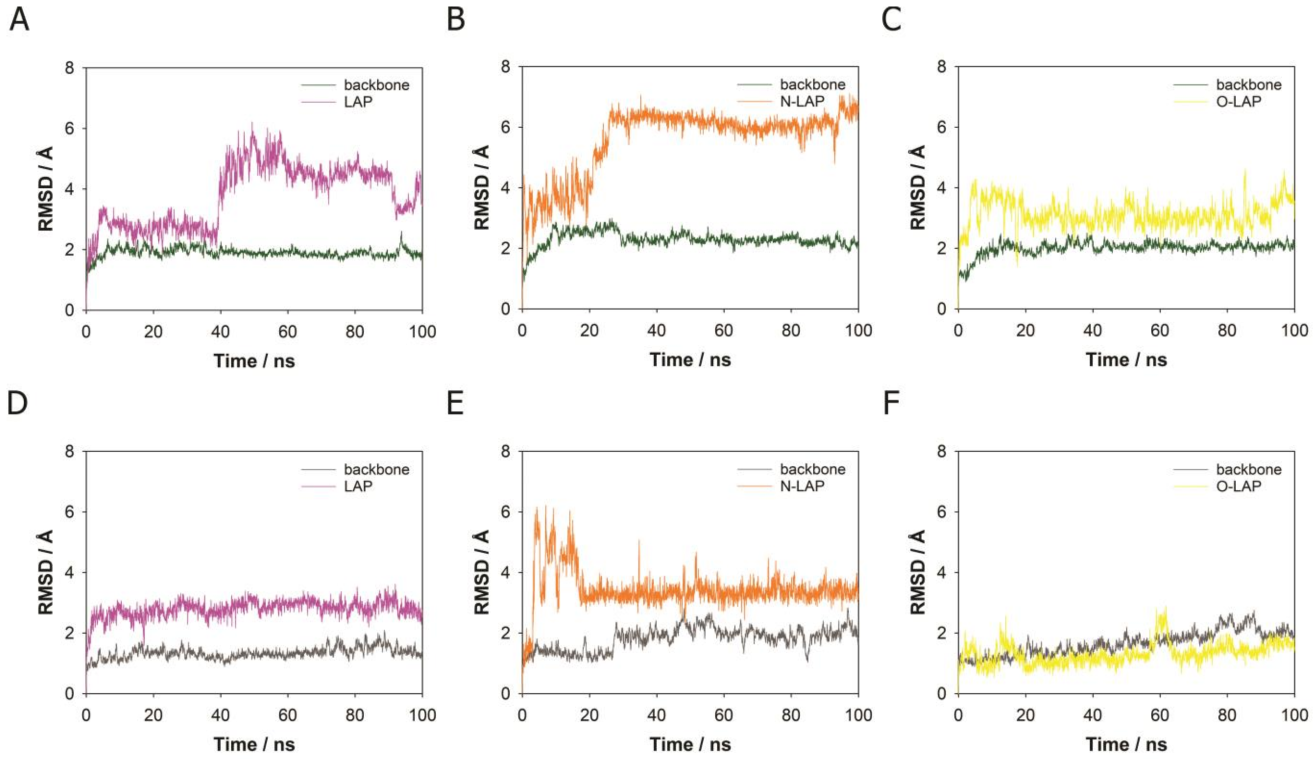

FIGURE S12. RMSD plots for the protein backbone (C$^{\alpha}$, C, N, and O atoms) calculated in the complexes LAP@BAG (A), N-LAP@BAG (B), O-LAP@BAG (C), LAP@HAG (D), N-LAP@HAG (E), and O-LAP@HAG (F) obtained from MD simulations studies. Green and grey lines are used for BAG and HAG proteins, respectively. Note how the rmsd values for the proteins are low and the conformation of the ligands is more flexible when binding to BAG than HAG.

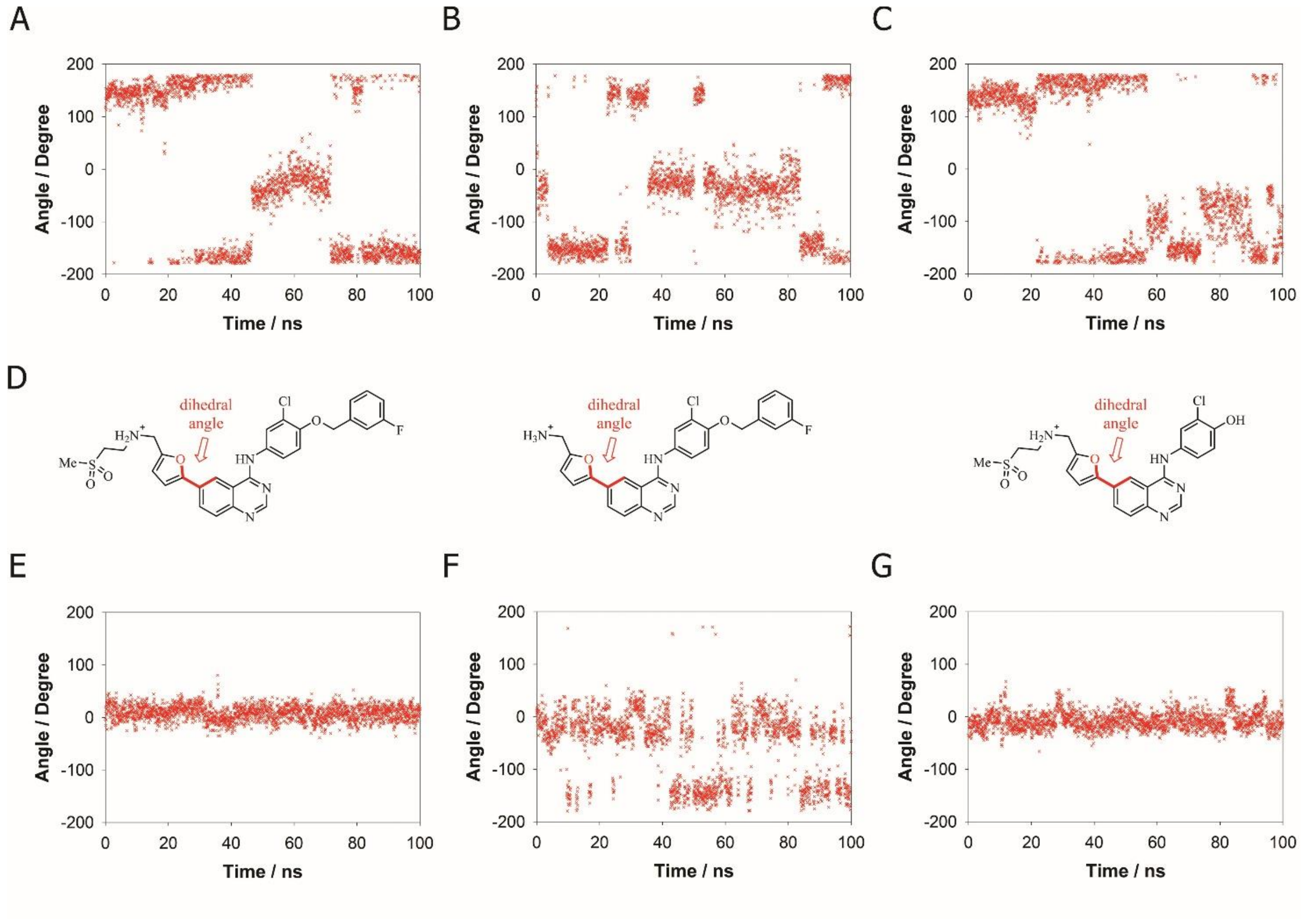

FIGURE S13. Variation of the dihedral angle between the furyl and quinazoline moieties of LAP, N-LAP and O-LAP in the LAP@BSA (A), N-LAP@BSA (B), O-LAP@BSA (C), LAP@HSA (E), N-LAP@HSA (F), and O-LAP@HSA (G) protein complexes during the whole simulation. The bonds involved in the dihedral angle are highlighted in red (D).

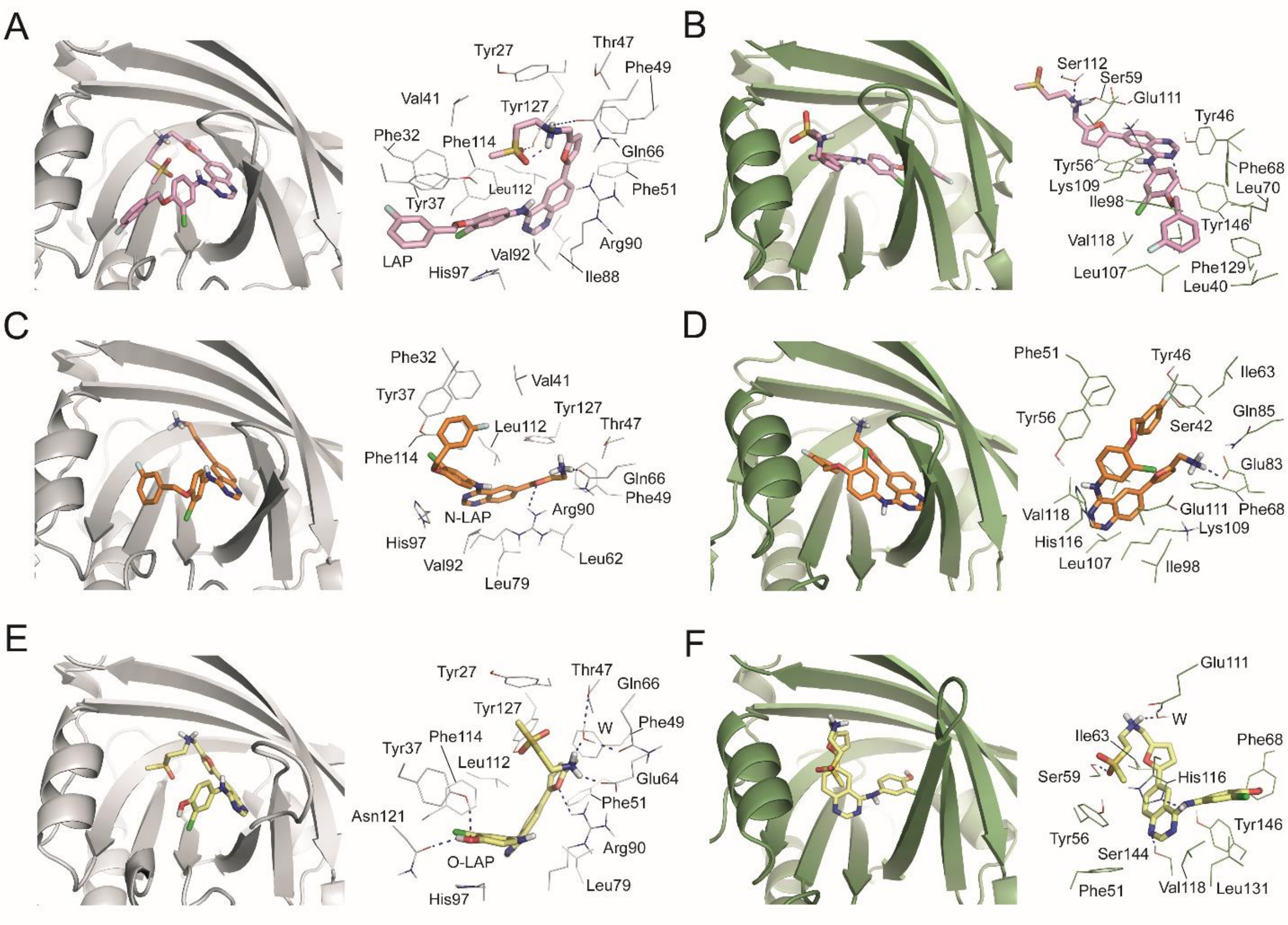

FIGURE S14. Proposed binding mode of LAP (pink, A-B), N-LAP (orange, C-D) and O-LAP (yellow, E-F) to HAG (gray) and BAG (green) as obtained by MD simulation studies. (A,C,E) Overall and detailed views of the proposed binary LAP@HAG, N-LAP@HAG and O-LAP@HAG complexes. (B,D,F) Overall and detailed views of the proposed binary LAP@BAG, N-LAP@BAG and O-LAP@BAG complexes. Snapshots after 80 ns are shown. Relevant side chain residues are shown and labelled. Electrostatic and hydrogen bonding interactions are shown as blue dashed lines.

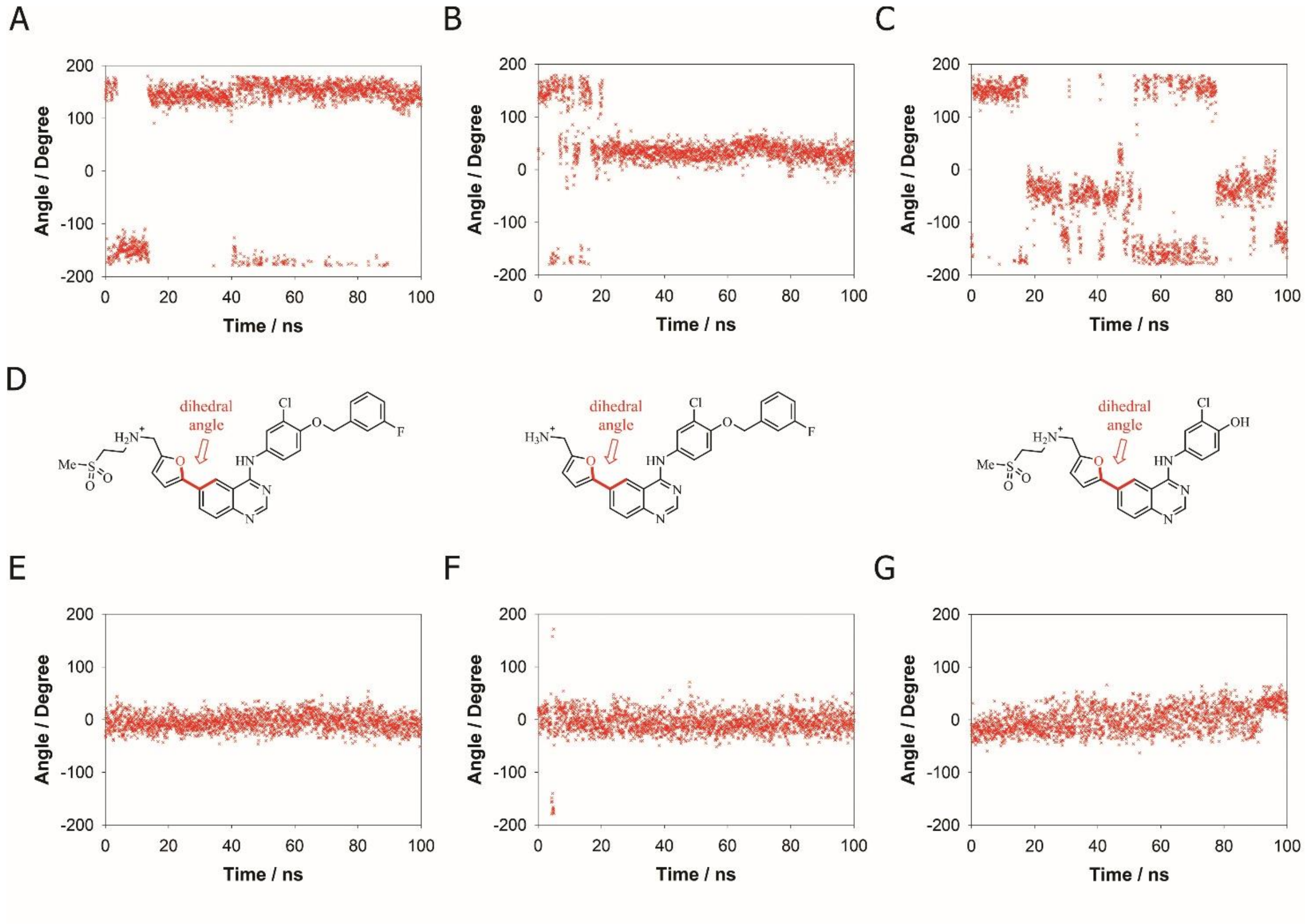

FIGURE S15. Variation of the dihedral angle between the furyl and quinazoline rings for LAP, N-LAP and O-LAP in the LAP@BAG (A), N-LAP@BAG (B), O-LAP@BAG (C), LAP@HAG (E), N-LAP@HAG (F), and O-LAP@HAG (G) complexes during the whole simulation. The bonds involved in the dihedral angle are highlighted in red (D).

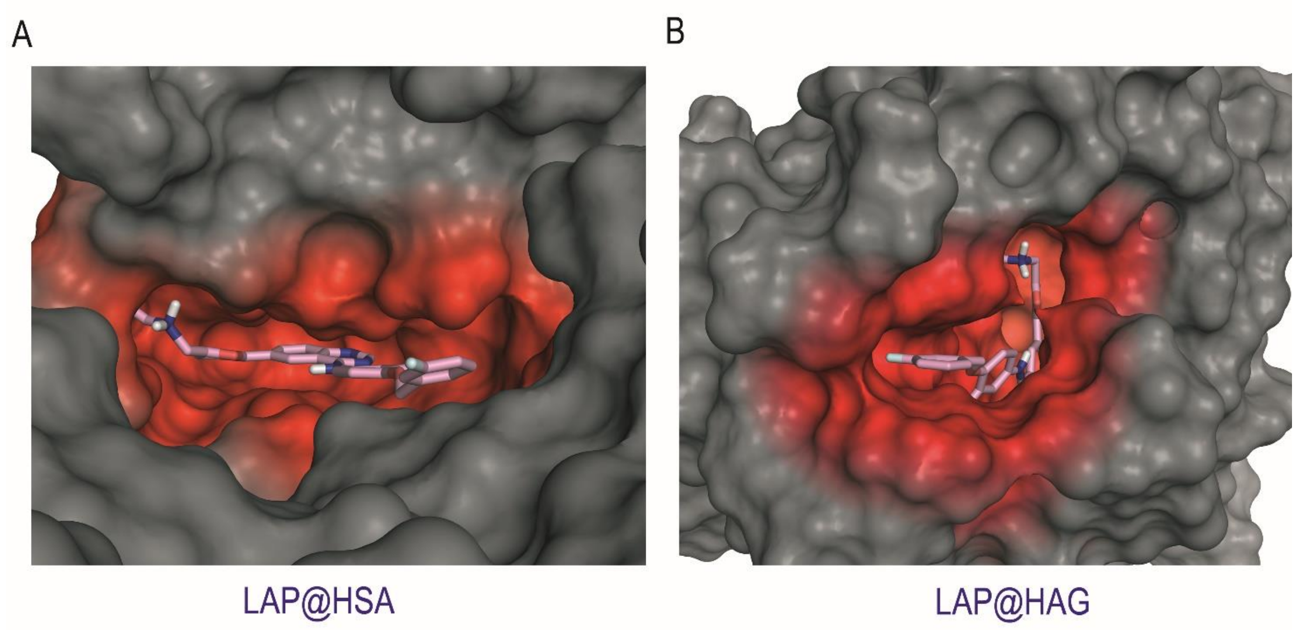

FIGURE S16. Close views of the proposed binding mode of LAP (pink) to HSA (A) and HAG (B) proteins as obtained by MD simulation studies. Snapshots after 80 ns are shown. The protein recognition center of the ligand is shown in red. Note how since the binding pocket of HSA is narrow while spherical in HAG, LAP is more covered by the protein in the LAP@HSA binary complex than in the LAP@HAG one.

```
HSA|      MKWVTFISLLFLFSSAYSRGVFRRDAHKSEVAHRFKDLGEENFKALVLIAFAQYLQQCPF
BSA|      ------------------------DTHKSEIAHRFKDLGEEHFKGLVLIAFSQYLQQCPF
                                  *:****:**********:**.******:********

HSA|      EDHVKLVNEVTEFAKTCVADESAENCDKSLHTLFGDKLCTVATLRETYGEMADCCAKQEP
BSA|      DEHVKLVNELTEFAKTCVADESHAGCEKSLHTLFGDELCKVASLRETYGDMADCCEKQEP
          ::*******:************  .*:*********:**.**:******:***** ****

HSA|      ERNECFLQHKDDNPNLPRLVRPEVDVMCTAFHDNEETFLKKYLYEIARRHPYFYAPELLF
BSA|      ERNECFLSHKDDSPDLPKL-KPDPNTLCDEFKADEKKFWGKYLYEIARRHPYFYAPELLY
          *******.****.*:**:* :*: :.:*  *: :*:.*  *******************:

HSA|      FAKRYKAAFTECCQAADKAACLLPKLDELRDEGKASSAKQRLKCASLQKFGERAFKAWAV
BSA|      YANKYNGVFQECCQAEDKGACLLPKIETMREKVLTSSARQRLRCASIQKFGERALKAWSV
          :*::*:..* ***** **.******:: :*::  :***:***:***:*******:***:*

HSA|      ARLSQRFPKAEFAEVSKLVTDLTKVHTECCHGDLLECADDRADLAKYICENQDSISSKLK
BSA|      ARLSQKFPKAEFVEVTKLVTDLTKVHKECCHGDLLECADDRADLAKYICDNQDTISSKLK
          *****:******.**:**********.**********************:***:******

HSA|      ECCEKPLLEKSHCIAEVENDEMPADLPSLAADFVESKDVCKNYAEAKDVFLGMFLYEYAR
BSA|      ECCDKPLLEKSHCIAEVEKDAIPENLPPLTADFAEDKDVCKNYQEAKDAFLGSFLYEYSR
          ***:**************:* :* :** *:***.*.******* ****.*** *****:*

HSA|      RHPDYSVVLLLRLAKTYETTLEKCCAAADPHECYAKVFDEFKPLVEEPQNLIKQNCELFE
BSA|      RHPEYAVSVLLRLAKEYEATLEECCAKDDPHACYSTVFDKLKHLVDEPQNLIKQNCDQFE
          ***:*:* :****** **:***:***  *** **:.***::* **:**********: **

HSA|      QLGEYKFQNALLVRYTKKVPQVSTPTLVEVSRNLGKVGSKCCKHPEAKRMPCAEDYLSVV
BSA|      KLGEYGFQNALIVRYTRKVPQVSTPTLVEVSRSLGKVGTRCCTKPESERMPCTEDYLSLI
          :**** *****:****:***************.*****::**.:**::****:*****::

HSA|      LNQLCVLHEKTPVSDRVTKCCTESLVNRRPCFSALEVDETYVPKEFNAETFTFHADICTL
BSA|      LNRLCVLHEKTPVSEKVTKCCTESLVNRRPCFSALTPDETYVPKAFDEKLFTFHADICTL
          **:***********::*******************  ******* *: : **********

HSA|      SEKERQIKKQTALVELVKHKPKATKEQLKAVMDDFAAFVEKCCKADDKETCFAEEGKKLV
BSA|      PDTEKQIKKQTALVELLKHKPKATEEQLKTVMENFVAFVDKCCAADDKEACFAVEGPKLV
          :.*:***********:*******:****:**::*.***:*** *****:*** ** ***

HSA|      AASQAALGL
BSA|      VSTQTALA-
          .::*:**.
```

FIGURE S17. Amino acid sequence alignments for the crystal structures of HSA (PDB code 4BKE) and BSA (4JK4). Protein sequences were aligned using the CLUSTAL Omega multiple sequence alignment (*http://www.ebi.ac.uk/Tools/msa/clustalo/, accessed July 24, 2020*). Subdomains IB for HSA (red) and BSA (blue) are highlighted. Note how the amino acid sequence of this part of the proteins is quite distinct (yellow shadow).

```
BAG:    MALLWALAVLSHLPLLDAQSPECANLMTVAPITNATMDLLSGKWFYIGSAFRNPEYKNSA
HAG:    ------------------EIPLCANLVP-VPITNATLDQITGKWFYIASAFRNEEYNKSV
                          : * ****:  .******:* ::******.***** **::*.

BAG:    RAIQAAFFYLEPRHAEDKLITREYQTIEDKCVYNCSFIKIYRQNGTLSKVESDREHFVDL
HAG:    QEIQATFFYFTPNKTEDTIFLREYQTRQDQCIYNTTYLNVQRENGTISRYVGGQEHFAHL
        : ***:***: *.::**.:: ***** :::*.** ::::: *:***:*:  . :**...*

BAG:    LLSKHFRTFMLAASWNGTKNVGVSFYADKPEVTQEQKKEFLDVIKCIGIQESEIIYTDEK
HAG:    LILRDTKTYMLAFDVNDEKNWGLSVYADKPETTKEQLGE---------------------
        *: :. :* *:.   :  ** *:*.******.*:**  *

BAG:    KDACGPLEKQHEEERKKETEASYEALDCLRIPRSDVMYTDWKKDKCEPLEKQHEKERKQE
HAG:    ---------------------FYEALDCLRIPKSDVVYTDWKKDKCEPLEKQHEKERKQE
                              **********:***:***********************
BAG:    -------------
HAG:    EGESAWSHPQFEK
```

FIGURE S18. Amino acid sequence alignments for the crystal structures of BAG homology model and HAG (3KQO). Protein sequences were aligned using the CLUSTAL Omega multiple sequence alignment (*http://www.ebi.ac.uk/Tools/msa/clustalo/, accessed July 24, 2020*). Note how the overall sequence similarity between both proteins is not high.

References

Vayá, I., Andreu, I., Lence, E., González-Bello, C., Cuquerella, M.C., Navarrete-Miguel, M., Roca-Sanjuán, D., and Miranda, M.A. (2020). Characterization of the locally excited and charge-transfer states of the anticancer drug lapatinib by ultrafast spectroscopy and computational studies. *Chem. Eur. J.* Accepted. DOI: 10.1002/chem.202001336

Vayá, I., Bonancia, P., Jimenez, M.C., Markovitsi, D., Gustavsson, T., and Miranda, M.A. (2013). Excited state interactions between flurbiprofen and tryptophan in drug-protein complexes and in model dyads. Fluorescence studies from the femtosecond to the nanosecond time domains. *Phys. Chem. Chem. Phys.* 15**,** 4727-4734. DOI: 10.1039/c3cp43847c
